# Supplementary material for: PPAR-γ regulates the effector function of human T helper 9 cells by promoting glycolysis
Source: Nat Commun. 2023 Apr 29;14:2471. doi: 10.1038/s41467-023-38233-x (PMC10148883; doi:10.1038/s41467-023-38233-x)
Supplement: Supplementary file 1 — Supplementary Information [file 41467_2023_38233_MOESM1_ESM.pdf]

PPAR-γ regulates the effector function of human T helper 9 cells by promoting glycolysis

Nicole L. Bertschi[1#], Oliver Steck[1#], Fabian Luther[1#], Cecilia Bazzini[1], Leonhard von Meyenn[1], Stefanie Schärli[1], Angela Vallone[1], Andrea Felser[2], Irene Keller[3], Olivier Friedli[4], Stefan Freigang[4], Nadja Begré[1], Susanne Radonjic-Hoesli[1], Cristina Lamos[1], Max Philip Gabutti[1], Michael Benzaquen[1], Markus Laimer[5], Dagmar Simon[1], Jean-Marc Nuoffer[2], Christoph Schlapbach[1*]

[1]Department of Dermatology, Inselspital, Bern University Hospital, University of Bern, Bern, Switzerland. [2]Institute of Clinical Chemistry, University of Bern, Bern, Switzerland. [3]Interfaculty Bioinformatics Unit and Swiss Institute of Bioinformatics, University of Bern, Bern, Switzerland. [4]Institute of Tissue Medicine and Pathology, University of Bern, Bern, Switzerland. [5]Department of Diabetes, Endocrinology, Nutritional Medicine and Metabolism (UDEM), Bern University Hospital, University of Bern, Bern, Switzerland.

[#]Contributed equally

*Correspondence and requests for materials should be addressed to C.S. (email: christoph.schlapbach@insel.ch)

Supplementary Figure 1

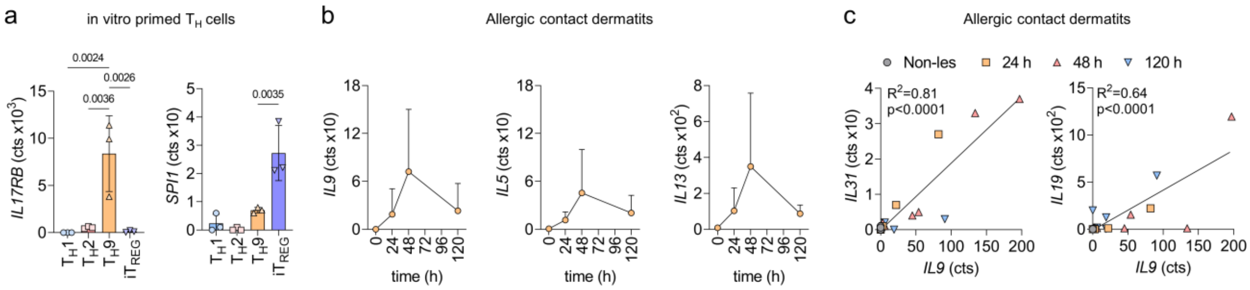

Supplementary Fig. 1: In vitro and in vivo primed T_H9 cells display key features of pathogenic T_H2 cells
a Expression levels of pT_H2-associated *IL17RB* and *SPI1* from the RNA-seq data shown in Fig. 1a. **b** *IL9, IL5, IL13* expression of untreated non-lesional (NL) skin and positive patch test reactions of lesional skin to nickel at 24 h, 48 h, and 120 h post allergen application from RNA-seq data shown in Fig. 1g-h. **c** In-sample correlations of *IL31* and *IL19* with *IL9* from RNA-seq data shown in Fig. 1g-h. The data are representative of independent experiments with three (a) or six (b, c) donors. Statistics: **a** One-way ANOVA, followed by a Dunnett's test for multiple comparisons. **c** Simple linear regression. The data are presented as mean ± SD.

Supplementary Figure 2

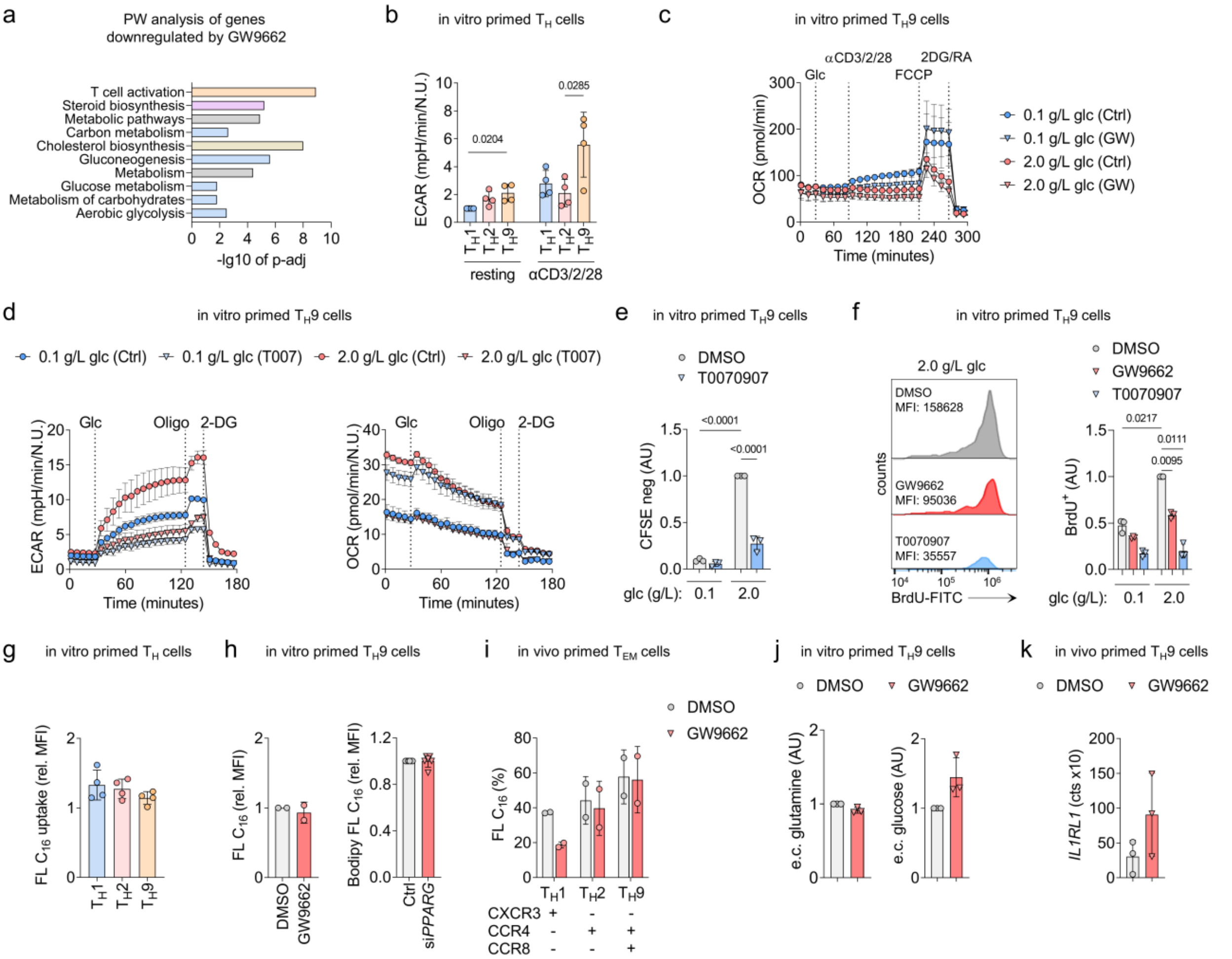

Supplementary Fig. 2: PPAR-γ mediates the high glycolytic activity of T_H9 cells

a Pathway analysis of downregulated genes in T_H9 clones in the presence of GW9662 activated by αCD3/CD2/CD28 for 12 h. **b** Basal acidification rate from data shown in Fig. 2c. **c** OCR measurement from data shown in Fig 2d. **d** OCR and ECAR measurements of in vitro primed T_H9 cells cultured in media with different glucose levels and GW9662 for 48 h and activated by injection of glucose and αCD3/CD2/CD28. **e** In vitro primed T_H9 cells were cultured in media with different glucose levels and activated with αCD3/CD2/CD28 for 4 d in presence of T0070907. Proliferation was measured by CFSE dilution using flow cytometry. **f** In vitro primed T_H9 cells were cultured in glucose of different levels for 48 h in presence of GW9662 and T0070907, respectively. Proliferation was measured 18 h after activation with αCD3/CD2/CD28 by BrdU using flow cytometry. **g** FA uptake by in vitro primed T_H cells measured with fluorescent BODIPY™FL C_{16} uptake by flow cytometry at day 7. **h** FA uptake by naïve T_H cells primed under T_H9 conditions for 7 days in the presence of GW9662 or transfected with *PPARG* and control siRNA, respectively. **i** FA uptake of in vivo primed effector memory T_H cells (T_{EM}) sorted by flow cytometry into T_H1, T_H2, and T_H9 cells according to their chemokine receptor profile and incubated in presence of GW9662 for 48 h. **j** Extracellular (e.c.) glutamine and glucose levels of naïve T_H cells primed under T_H9 conditions for 7 days in presence of GW9662. **k** Expression levels of *IL1RL1* from the RNA-seq data of T_H9 clones in the presence of GW9662 activated by αCD3/CD2/CD28 for 12 h. The data are representative of one experiment with three clones from one donor (a), one experiment with three donors (f), or independent experiments with two (h left, i), three (c, e, j), four (b, g) or six (d, h right) donors. Statistics: **a** Fisher's one-tailed test. **h, j, k** Two-tailed paired t-test. **e** One-way ANOVA, followed by a Šidák's test for multiple comparisons. **b, f, g** One-way ANOVA, followed by a Tukey's test for multiple comparisons. **i** Two-way ANOVA, followed by a Tukey's test for multiple comparisons. The data are presented as mean ± SD.

Supplementary Figure 3

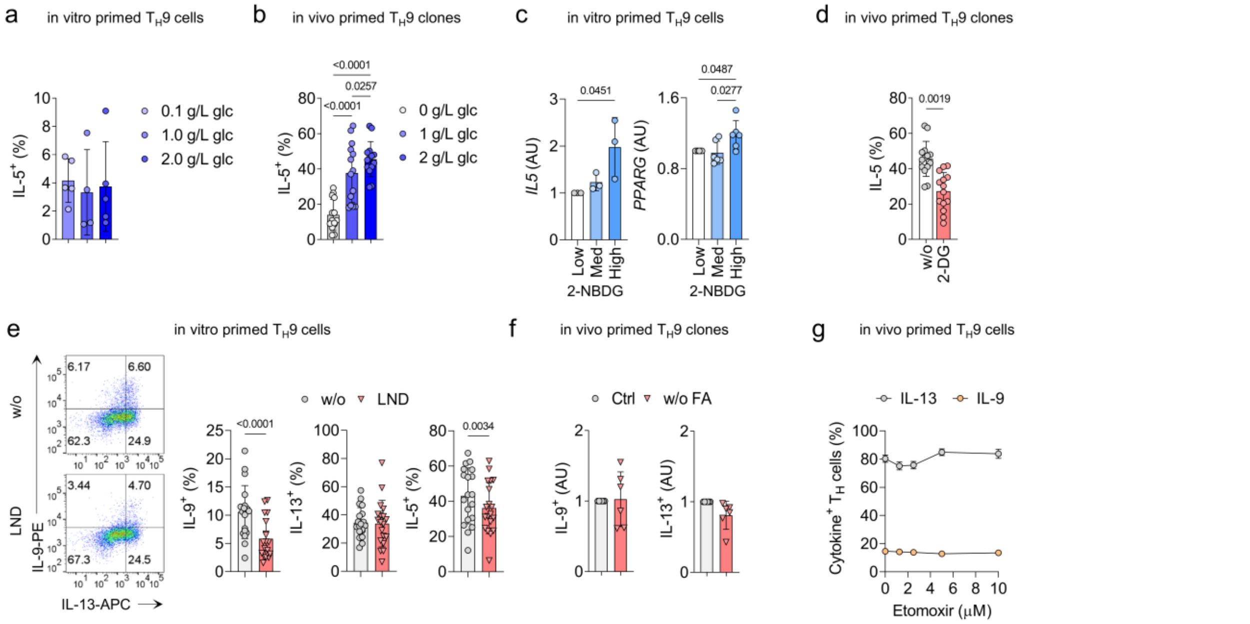

Supplementary Fig. 3: High glycolytic activity of T_H9 cells regulates specific effector functions

a IL-5 expression measured by flow cytometry of T_H9 cells primed in vitro in media containing glucose of different levels for 7 d, corresponding to data from Fig. 3a. **b** IL-5 expression of in vivo primed T_H9 clones cultured for 72 h in media containing glucose of different levels, corresponding to data from Fig. 3b. **c** In vitro primed T_H9 cells were activated for 4 h with αCD3/CD2/CD28, then sorted by flow cytometry based on their glucose uptake measured by 2-NBDG uptake. *IL5* and *PPARG* expression in the sorted T_H cell populations measured by RT-qPCR. Data corresponds to Fig. 3c. **d** IL-5 expression of in vivo primed T_H9 cells cultured for 7 days in the presence of 2-DG, corresponding to data from Fig. 3d. **e** Cytokine expression of in vitro primed T_H9 cells cultured in presence of lonidamine (LND), measured by flow cytometry 24 h after activation with αCD3/CD2/CD28. **f** In vivo primed T_H9 clones were cultured in media without fatty acid. Cytokines were measured 18 h after activation with αCD3/CD2/CD28. **g** In vivo primed T_H9 clones were cultured with etomoxir for 48 h. Cytokines were measured 18 h after activation with αCD3/CD2/CD28. The data are representative of independent experiments with three (c) or five (a) donors or six (f), fourteen (b, d) or eighteen (e) clones from two donors or three clones from one donor (g). Statistics: **a, c** One-way ANOVA, followed by a Tukey's test for multiple comparisons. **b** One-way ANOVA, followed by a Šidák's test for multiple comparisons **d-f** Two-tailed paired t-test. The data are presented as mean ± SD.

Supplementary Figure 4

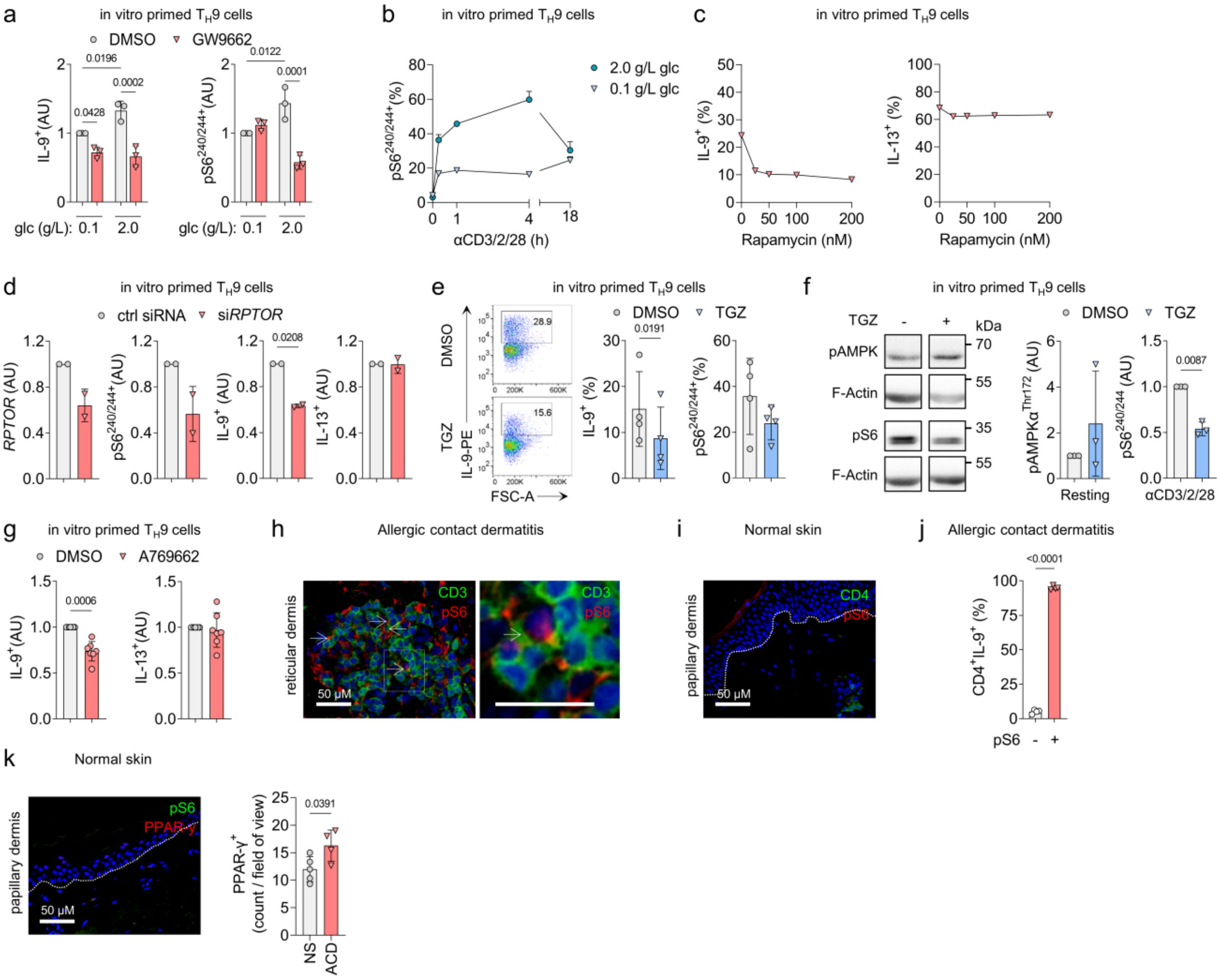

Supplementary Fig. 4: mTORC1 integrates bioenergetics with effector function in T_H9 cells

a Corresponding data shown in Fig. 4a. Cells were cultured for 48 h in glucose of different levels in the presence of GW9662. Cytokine expression and pS6 were measured by flow cytometry 18 h after activation with αCD3/CD2/CD28. **b** In vitro primed T_H9 cells were cultured in glucose of different levels for 48 h and activated with αCD3/CD2/CD28. pS6 was measured by flow cytometry at different time points. **c** In vitro primed T_H9 cells were cultured with rapamycin of different concentrations for 48 h, and cytokine expression was measured as in (a). **d** In vitro primed T_H9 cells were transfected with *RPTOR* and control siRNA. After 12 h, *RPTOR* expression was measured by RT-qPCR. Cytokine expression and pS6 were measured by flow cytometry after 18 h after activation with αCD3/CD2/CD28. **e** In vitro primed T_H9 cells were incubated in presence of troglitazone (TGZ) for 48 h. Cytokine expression and pS6 were measured by flow cytometry 18 h after activation with αCD3/CD2/CD28. **f** In vitro primed T_H9 cells were incubated with TGZ for 48 h. pAMPK levels and pS6 levels were measured by Western blot in the resting state and 18 h after activation with αCD3/CD2/CD28, respectively. **g** In vitro primed T_H9 cells were incubated with A769662 for 48 h and cytokine expression were measured by flow cytometry 18 h after activation with αCD3/CD2/CD28. **h** Immunofluorescence staining for CD3 and pS6 on skin samples of ACD. Scale bars, 50μM. **i** Immunofluorescence staining for CD4 and pS6 on normal skin (NS) samples. Scale bars, 50μM. **j** Percentage of $pS6^-$ and $pS6^+$ cells in $CD4^+IL\text{-}9^+$ T_H cells isolated from ACD. **k** Immunofluorescence staining for pS6 and PPAR-γ on normal skin samples (NS) and quantification of PPAR-γ^+ cells in NS and ACD skin samples. Scale bars, 50μM. The data are representative of one experiment with two (d) or three donors (f), independent experiments with one (j), two (b), three (a), four (e, k (ACD)), five (c, h, i, k (NS)) or seven (g) donors. Statistics: **a** One-way ANOVA, followed by a Šidák's test for multiple comparisons. **d-g** Two-tailed paired t-test. **j, k** Two-tailed unpaired t-test. The data are presented as mean ± SD.

Supplementary Figure 5

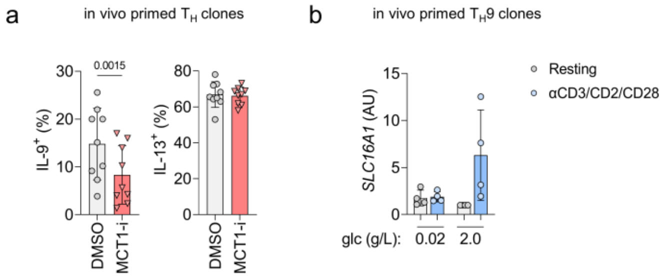

Supplementary Fig. 5: Paracrine IL-9 promotes aerobic glycolysis in IL-9R⁺ T_H cells by inducing the lactate transporter MCT1

a In vivo primed IL-9R⁺ T_H clones were pre-incubated with the MCT1 inhibitor (MCT1-i) BAY-8002 for 1 h. Cytokine expression measured 18 h after activation with αCD3/CD2/CD28 by flow cytometry. **b** In vivo primed IL-9R⁺ T_H clones were cultured in media containing glucose of different levels for 3 days. RNA expression of *SLC16A1* was measured by RT-qPCR in the resting state or 24 h after activation with αCD3/CD2/CD28. The data are representative of one experiment with two clones from two donors (b) or independent experiments with nine clones from two donors (a). Statistics: **a** Two-tailed paired t-test. **b** One-way ANOVA, followed by a Tukey's test for multiple comparisons. The data are presented as mean ± SD.

Supplementary Figure 6

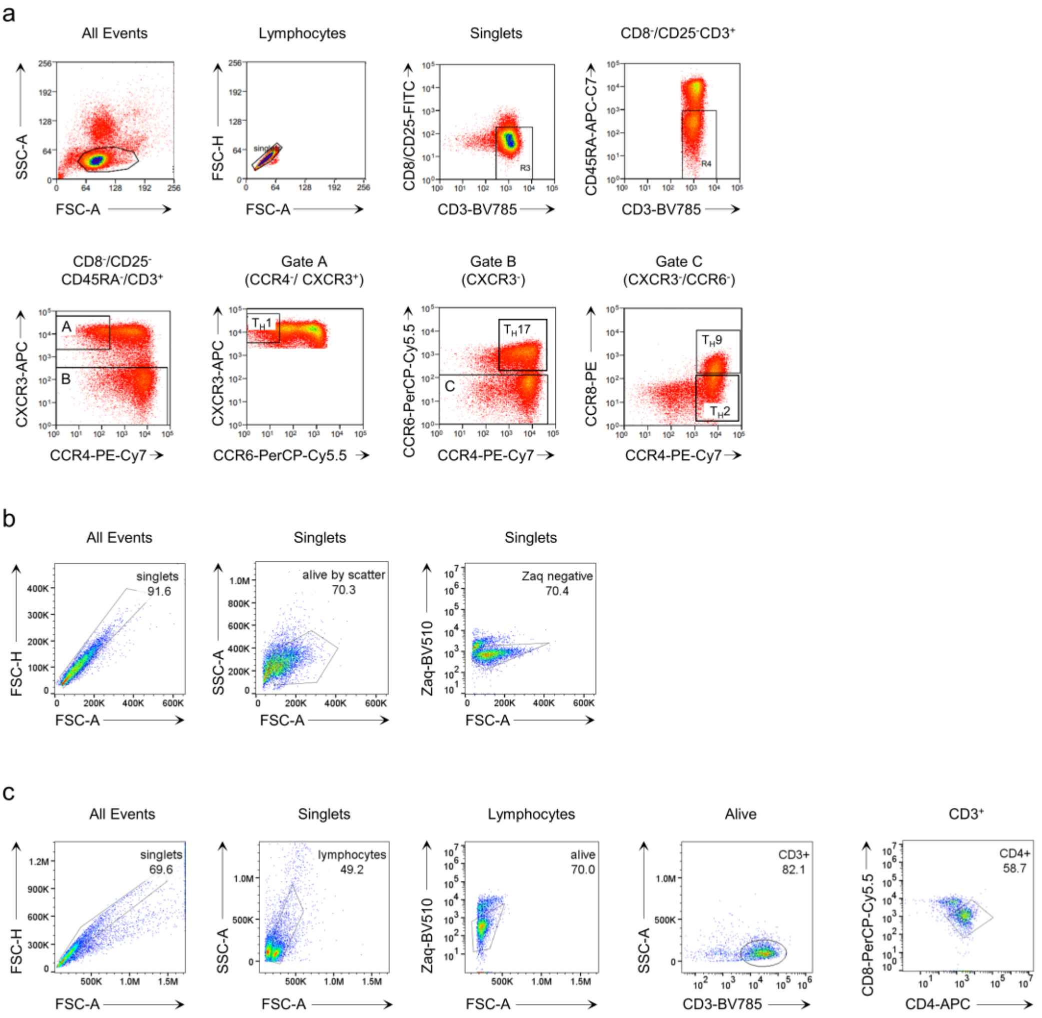

Supplementary Fig. 6: Gating strategies for cell sorting and flow cytometry

a Gating strategy for T_H cell subset clones sorting for Fig. 2b, 2h, 2k, 3b, 3d, 5a-b, e-m, 6a-c and Supplementary Fig. 2i, 3b, 3d, 3f-g, 5a. CD4$^+$ T cells were isolated from PBMC of healthy donors stained for the subsequent sorting of the T_H cell subset. Memory T_H cell subsets were sorted according to the expression of chemokine receptors from CD45RA$^-$CD25$^-$CD8$^-$CD3$^+$ cells: T_H1 (CXCR3$^+$CCR8$^-$CCR6$^-$CCR4$^-$), T_H2 (CXCR3$^-$CCR8$^-$CCR6$^-$CCR4$^+$), T_H9 (CXCR3$^-$CCR8$^+$CCR6$^-$CCR4$^+$), and T_H17 (CXCR3$^-$CCR8$^-$CCR6$^+$CCR4$^+$). **b** Gating strategy for Fig. 2f-i, 3a-e, 4b-e, 5a-b, 6a-c and Supplementary Fig. 2e-i, 3a-g, 4a-e, 4g, 5a of in vitro primed T_H cell subsets or FACS sorted in vivo primed T_H cell subsets. Cells were gated based on FSC height and FSC area for singlets, either zombie aqua (Zaq) negative or SSC and FCS for live cells. Live cells were analyzed for glucose and fatty acid uptake, cytokine expression or phosphorylation of the target of interest. **c** Gating strategy for Fig. 4g, 5d and Supplementary Fig. 4j of T_H cells isolated from skin biopsies. Cells were gated based on FSC height and FSC area for singlets, SSC and FCS for lymphocytes, zombie aqua (Zaq) negative for live cells and further gated for CD3$^+$ and CD4$^+$ or CD8$^+$ cells. CD4$^+$ cells were analyzed for cytokine expression or phosphorylation of the target of interest.

Supplementary Tables

Table S1: Antibodies, recombinant proteins and chemicals used in this study

Flow cytometry - Surface staining	Antibody	Clone	Conjugation	Company (Article number)	Dilution
	Mouse anti-human CXCR3, mAb	G025H7	AF647	BioLegend (353712)	1:60
			FITC	BioLegend (353704)	1:200
	Mouse anti-human CD45RA, mAb	HI100	APC-Cy7	BioLegend (304128)	1:200
			PerCP-Cy5.5	BioLegend (304122)	1:1600
	Mouse anti-human CD8, mAb	RPA-T8	FITC	BioLegend (301021)	1:100
			PerCP-Cy5.5	BioLegend (344710)	1:1600
			BV660	BioLegend (301042)	1:400
	Mouse anti-human CD25, mAb	BC96	FITC	BioLegend (302604)	1:100
	Mouse anti-human CCR8, mAb	L263G8	PE	BioLegend (360604)	1:60
			APC	BioLegend (360609)	1:200
	Mouse anti-human CCR7, mAb	G043H7	PE-Cy7	BioLegend (353226)	1:200
	Mouse anti-human CCR4, mAb	L291H4	PE-Cy7	BioLegend (359410)	1:60
			BV605	BioLegend (359417)	1:200
	Mouse anti-human CCR6, mAb	G034E3	PerCP-Cy5.5	BioLegend (353406)	1:60
			BV421	BioLegend (353407)	1:200
	Mouse anti-human CD3, mAb	OKT3	BV785	BioLegend (317330)	1:200
	Mouse anti-human CD4, mAb	OKT4	APC-Cy7	BioLegend (317418)	1:400
	Mouse anti-human IL-9R, mAb	AH9R7	PE	BioLegend (310404)	1:200
	Mouse anti-human IgG2b, mAb	MG2b-57	PE	BioLegend (401207)	1:200
	2-NBDG		FITC	Cayman Chemicals (11046)	1 ng/ml
	CFSE		FITC	Selleckchem (S8269)	2 µM

	BODIPY™FL C_{16}		FITC	Thermo Fisher Scientific (D3821)	20 nM
	Phase-Flow™ FITC BrdU Kit		FITC	BioLegend (370704)	1:100
Flow cytometry – Intracellular staining	Antibody	Clone	Conjugation	Company	Dilution
	Rat anti-human IL-4, mAb	MP4-25D2	PE-Cy7	BioLegend (500824)	1:400
	Rat anti-human IL-5, mAb	TRFK5	BV421	BioLegend (504311)	1:400
	Mouse anti-human IL-9, mAb	MH9A4	PE	BioLegend (507605)	1:400
	Rat anti-human IL-13, mAb	JES10-5A2	APC	BioLegend (501907)	1:300
	Mouse anti-human INF-γ, mAb	B27	PE-Cy7	BioLegend (506518)	1:400
	Rabbit anti-human pS6, mAb	D57.2.2E	AF488	Cell Signaling (4803S)	1:800
Immunofluorescence Assays	Antibody	Clone	Conjugation	Company	Dilution
	Mouse anti-human CD3, mAb	F7.2.38	unconjugated	Dako (M7254)	1:50
	Mouse anti-human CD4, mAb	4B12	unconjugated	Novocastra (NCL-L-CD4-368)	1:50
	Mouse anti-human PPAR-γ, mAb	E8	unconjugated	Santa Cruz (sc-7273)	1:100
	Rabbit anti-human pS6, mAb	D57.2.2E	unconjugated	Cell Signaling (4858S)	1:100
	Goat anti-rabbit IgG, pAb		AF488	Invitrogen (A11008)	1:500
	Goat anti-rabbit IgG, mAb		AF594	Invitrogen (A11072)	1:500
	Goat anti-mouse IgG1, mAb		AF594	Invitrogen (A21125)	1:500
	Fluoromount G with DAPI			Southern Biotech (0100-20)	
Western Blotting	Antibody	Clone	Conjugation	Company	Dilution
	Rabbit anti-human PPAR-γ, mAb	C26H12	unconjugated	Cell Signaling (2435)	1:500
	Rabbit anti-human pS6, mAb	D57.2.2E	unconjugated	Cell Signaling (4858S)	1:2000
	Rabbit anti-human pAMPK, mAb	40H9	unconjugated	Cell Signaling (2535S)	1:1000
	Mouse anti-human MCT1, mAb	H-1	unconjugated	Santa Cruz (sc-365501)	1:250
	Rabbit anti-human Histone H2B, pAb		unconjugated	Sigma-Aldrich (SAB4502231)	1:1000

	Mouse anti-human F-Actin, mAb	ACTN05	unconjugated	Invitrogen (MA5-11869)	1:6000
	Goat anti-mouse IgG, pAb		HRP	Thermo Fisher Scientific (G21040)	1:5000
	Goat anti-rabbit IgG, pAb		HRP	Fisher scientific (31462)	1:5000
Recombinant proteins & Chemicals	Name		Format	Company	Final Concentration
	rhIL-2		purified	Hoffmann-La Roche	50/250 IU/ml
	rhIL-4		purified	BioLegend (574006)	50 ng/ml
	rhTGF-beta		purified	R&D (240-B-010)	5 ng/ml
	rhIL-12		purified	BioLegend (573004)	5 ng/ml
	rhIL-9		purified	BioLegend (594404)	5 ng/ml
	GW9662			Sigma-Aldrich (M6191)	10 µM
	T0070907			Selleckchem (S2871)	10 µM
	Rapamycin			Sigma-Aldrich (R0395)	50 nM
	BAY-8002			Selleckchem (S8747)	75 µM
	2-DG			Sigma-Aldrich (D8375)	1 mM
	Lonidamine			Sigma-Aldrich (L4900)	100 µM
	Troglitazone			Selleckchem (S8432)	25 µM
	Etomoxir			Selleckchem (S8244)	1-10 µM
	A769662			Selleckchem (S2697)	10 µM
	Ritlecitinib			Selleckchem (S8538)	100 nM

Table S2: Quantitative reverse transcription-polymerase chain reaction (RT-qPCR) primers used in the study

Gene Transcript	Gene	Species	TaqMan Primer (Thermo Fisher Scientific)
IL-4	*IL4*	human	Hs00174122_m1
IL-5	*IL5*	human	Hs01548712_g1
IL-9	*IL9*	human	Hs00174125_m1
IL-13	*IL13*	human	Hs00174379_m1
RPTOR	*RPTOR*	human	Hs00375332_m1
MCT1	*SLC16A1*	human	Hs01560299_m1
PPAR-γ	*PPARG*	human	Hs01115513_m1
HPRT1	*HPRT1*	human	Hs99999909_m1

Table S3: Allergens from Figure 6D

Sample ID	Allergen	Intensity of Reaction/<br>biopsy collection post allergen application (hours)
1	Perubalsam	+++ / 48 h
2	p-Phenylendiamin	+++ / 48 h
3	Primin	+++ / 48 h
4	Propolis	+++ / 48 h
5	p-Phenylendiamin	+++ / 48 h
6	p-Phenylendiamin	+++ / 48 h

Table S4: siRNA used in the study

Gene transcript	Gene	Species	siRNA (Company)	Cat. Number
PPAR-γ	*PPARG*	human	Silencer™ Select *PPARG* siRNA (Thermo Fisher Scientific)	s10886<br>s10887<br>s10888
MCT1	*SLC16A1*	human	Silencer™ Select *SLC16A1* siRNA (Thermo Fisher Scientific)	s579<br>s580
			MCT1 siRNA (Santa Cruz)	sc-37235
RPTOR	*RPTOR*	human	RPTOR siRNA (Santa Cruz)	sc-44069
negative control	-	human	Silencer™ Select Negative Control No. 1 siRNA (Thermo Fisher Scientific)	4390843
