# Supplementary material for: PPAR-γ regulates the effector function of human T helper 9 cells by promoting glycolysis
Source: Nat Commun. 2023 Apr 29;14:2471. doi: 10.1038/s41467-023-38233-x (PMC10148883; doi:10.1038/s41467-023-38233-x)
Supplement: Supplementary file 3 — Reporting Summary [file 41467_2023_38233_MOESM3_ESM.pdf]

nature portfolio

Corresponding author(s): Nicole L. Bertschi, Christoph Schlapbach

Last updated by author(s): Apr 3, 2023

Reporting Summary

Nature Portfolio wishes to improve the reproducibility of the work that we publish. This form provides structure for consistency and transparency in reporting. For further information on Nature Portfolio policies, see our Editorial Policies and the Editorial Policy Checklist.

Statistics

For all statistical analyses, confirm that the following items are present in the figure legend, table legend, main text, or Methods section.

n/a	Confirmed	
☐	☒	The exact sample size (*n*) for each experimental group/condition, given as a discrete number and unit of measurement
☐	☒	A statement on whether measurements were taken from distinct samples or whether the same sample was measured repeatedly
☐	☒	The statistical test(s) used AND whether they are one- or two-sided<br>*Only common tests should be described solely by name; describe more complex techniques in the Methods section.*
☒	☐	A description of all covariates tested
☐	☒	A description of any assumptions or corrections, such as tests of normality and adjustment for multiple comparisons
☐	☒	A full description of the statistical parameters including central tendency (e.g. means) or other basic estimates (e.g. regression coefficient) AND variation (e.g. standard deviation) or associated estimates of uncertainty (e.g. confidence intervals)
☐	☒	For null hypothesis testing, the test statistic (e.g. *F*, *t*, *r*) with confidence intervals, effect sizes, degrees of freedom and *P* value noted<br>*Give P values as exact values whenever suitable.*
☒	☐	For Bayesian analysis, information on the choice of priors and Markov chain Monte Carlo settings
☒	☐	For hierarchical and complex designs, identification of the appropriate level for tests and full reporting of outcomes
☒	☐	Estimates of effect sizes (e.g. Cohen's *d*, Pearson's *r*), indicating how they were calculated

Our web collection on statistics for biologists contains articles on many of the points above.

Software and code

Policy information about availability of computer code

Data collection

1. Flow cytometry data were collected on CytoFLEX (Beckman Coulter) with CytExpert software version 2.4 (Beckman Coulter)
2. Cells were sorted using the MoFlo ASTRIOS EQ with Summit software version 6.3.1 (Beckman Coulter)
3. qRT-PCR data were collected on a 7300 Real-Time PCR System (Applied Biosystems) and analysed using the Sequence Detection Software version 1.4 (Applied Biosystems)
4. RNAseq data was collected on an Illumina HiSeq3000
5. Seahorse analysis was performed on a Seahorse XFe96 Analyzer (Seahorse Biosciences, Agilent Technologies) using the Wave software version 2.6.3 (Agilent Technologies)
6. Western blots were visualized by the Fusion Pulse TS of Vilber Lourmat (Witec) using the Evolution Capt Pulse 6 software version 17.02
7. Lumiesence and Absorbance was analysed by Tecan Reader Spark 10M using the Tecan Spark Control Software version 1.2
8. Immunofluorescence images were acquired on an Eclipse Microscope (Nikon) using the NIS Elements Imaging Software version 4.2 (Nikon)

Data analysis	1. Flow cytometry data were analysed with CytExpert software version 2.4 (Beckman Coulter) and the FlowJo software version 10 (BD Life Sciences)<br>2. RNAseq analysis: The RNAseq reads were mapped to the reference human genome (GRCh38, build 81) using HISAT2 version 2.0.4. HTseq-count version 0.6.1 was used to count the number of reads per gene, and DESeq2 version 1.4.5 was used to test for differential expression between groups of samples.<br>3. Pathway analysis was performed using the g:Profiler (ELIXIR infrastructure), version e108_eg55_p17_9f356ae, updated 2022<br>4. Quantification of Western blots was performed on ImageJ version 1.52a<br>5. All the statistical analysis were performed on Prims version 9 (GraphPad)

For manuscripts utilizing custom algorithms or software that are central to the research but not yet described in published literature, software must be made available to editors and reviewers. We strongly encourage code deposition in a community repository (e.g. GitHub). See the Nature Portfolio guidelines for submitting code & software for further information.

Data

Policy information about availability of data

All manuscripts must include a data availability statement. This statement should provide the following information, where applicable:

- Accession codes, unique identifiers, or web links for publicly available datasets
- A description of any restrictions on data availability
- For clinical datasets or third party data, please ensure that the statement adheres to our policy

The RNAseq data of this study have been deposited in BioStudios with the accession codes E-MTAB-12204 [https://www.ebi.ac.uk/biostudies/arrayexpress/studies/E-MTAB-12204], E-MTAB-12199 [https://www.ebi.ac.uk/biostudies/arrayexpress/studies/E-MTAB-12199], E-MTAB-12237 [https://www.ebi.ac.uk/biostudies/arrayexpress/studies/E-MTAB-12237] and E-MTAB-12197 [https://www.ebi.ac.uk/biostudies/arrayexpress/studies/E-MTAB-12197]. Publicly available data with accession code GSE93219 [https://www.omicsdi.org/dataset/geo/GSE93219], GSE130148 [https://www.omicsdi.org/dataset/geo/GSE130148], GSE175930 [https://www.omicsdi.org/dataset/omics_ena_project/PRJNA734315] and E-MTAB-5739 [https://www.ebi.ac.uk/biostudies/arrayexpress/studies/E-MTAB-5739] were re-analyzed. The RNAseq reads were mapped to the reference human genome GRCh38, build 81 [https://www.ncbi.nlm.nih.gov/assembly/GCF_000001405.26/]. The authors declare that all other data supporting the finding of this study are available within the article and its supplementary information files. Source data for figures 1-6 and supplementary figures 1-5 are provided with the paper.

Human research participants

Policy information about studies involving human research participants and Sex and Gender in Research.

Reporting on sex and gender	Both male and female participants were included.
Population characteristics	All the participants were aged between 25-74 years with no past history of disease or illness.
Recruitment	Participants were selected randomly and in an unbiased manner. The skin was obtained from healthy patients who underwent cosmetic surgery procedures or patients with ACD, or positive patch test reactions to standard contact allergens. Volunteers were well informed and their written consent was taken prior to sample collection.
Ethics oversight	All experiments performed on human tissue samples were conducted in accordance with the Declaration of Helsinki. Human blood was obtained from healthy donors from the Swiss Blood Donation Center in Bern and was used in compliance with the Federal Office of Public Health (authorization no. P_149). The study on human patient samples was approved by the Medical Ethics Committee of the Canton of Bern, Switzerland (no. 088/13; 2019-01068; 2019-00803). Written informed consent was obtained from all patients.

Note that full information on the approval of the study protocol must also be provided in the manuscript.

Field-specific reporting

Please select the one below that is the best fit for your research. If you are not sure, read the appropriate sections before making your selection.

☒ Life sciences ☐ Behavioural & social sciences ☐ Ecological, evolutionary & environmental sciences

For a reference copy of the document with all sections, see nature.com/documents/nr-reporting-summary-flat.pdf

Life sciences study design

All studies must disclose on these points even when the disclosure is negative.

Sample size	No statistical power test were used to determine sample size needed. At least three biological replicates (except Figure 4G) were analyzed and different donors were used to account for variation. The exact n numbers used in each experiment are indicated in the figure legends.
Data exclusions	No data were excluded from the analyses.
Replication	Experimental findings were reliably reproduced within our lab. Results shown are technical replicates from representative biological

	replicates. The information of replication is clearly found in the Figure legends.
Randomization	Each sample of blood and human tissue cells were split into two and treated with the respective compound or left untreated (control).
Blinding	Studies on blood and human tissue cells were performed without blinding. Data were analyzed by software with objective outcomes and quantification was performed in an uniform manner for all samples tested. Therefore, blinding was not relevant for this study.

Reporting for specific materials, systems and methods

We require information from authors about some types of materials, experimental systems and methods used in many studies. Here, indicate whether each material, system or method listed is relevant to your study. If you are not sure if a list item applies to your research, read the appropriate section before selecting a response.

Materials & experimental systems

n/a	Involved in the study	
☐	☒	Antibodies
☒	☐	Eukaryotic cell lines
☒	☐	Palaeontology and archaeology
☒	☐	Animals and other organisms
☒	☐	Clinical data
☒	☐	Dual use research of concern

Methods

n/a	Involved in the study	
☒	☐	ChIP-seq
☐	☒	Flow cytometry
☒	☐	MRI-based neuroimaging

Antibodies

Antibodies used

Flow cytometry:
(Target; Clone; Brand; Fluorochrome and corresponding Catalog number)
mouse anti-human CXCR3; G025H7; BioLegend; AF647 Cat #353712; FITC Cat# 353704
mouse anti-human CD45RA; HI100; BioLegend; APC-Cy7 Cat# 304128; PerCP-Cy5.5 Cat#304122
mouse anti-human CD8; RPA-T8; BioLegend; FITC Cat#301021; PerCP-Cy5.5 Cat#344710; BV660 Cat#301042
mouse anti-human CD25; BC96; BioLegend; FITC Cat#302604
mouse anti-human CCR8; L263G8; BioLegend; PE Cat#360604; APC Cat#360609
mouse anti-human CCR7; G043H7; BioLegend; PE-Cy7 Cat#353226
mouse anti-human CCR4; L291H4; BioLegend; PE-Cy7 Cat#359410; BV605 Cat#359417
mouse anti-human CCR6; G034E3; BioLegend; PerCP-Cy5.5 Cat#353406; BV421 Cat#353407
mouse anti-human CD3; OKT3; BioLegend; BV785 Cat#317330
mouse anti-human CD4; OKT4; BioLegend; APC-Cy7 Cat#317418
mouse anti-human IL-9R; AH9R7; BioLegend; PE Cat#310404
mouse anti-human IgG2b; MG2b-57; BioLegend; PE Cat#401207
rat anti-human IL-4; MP4-25D2; BioLegend; PE-Cy7 Cat#500824
rat anti-human IL-5; TRFK5; BioLegend; BV421 Cat#504311
mouse anti-human IL-9; MH9A4; BioLegend; PE Cat#507605
rat anti-human IL-13; JES10-5A2; BioLegend; APC Cat# PE-Cy7 Cat#506518
rabbit anti-human pS6; D57.2.2E; Cell Signaling; AF488 Cat#4803S
Phase-Flow™ FITC anti-BrdU; 3D4; BioLegend; FITC Cat#370704

Immunofluorescence
Primary Antibodies (Target; Clone; Brand; Fluorochorme and corresponding catalog number)
mouse anti-human CD3; 7.2.38; Dako; unconjugated; Cat#M7254
mouse anti-human CD4 ;4B12; Novocastra; unconjugated Cat#NCL-L-CD4-368
mouse anti-human PPAR-γ; E8; Santa Cruz; unconjugated Cat#sc-7273
rabbit anti-human pS6; D57.2.2E; Cell Signaling; unconjugated Cat#4858S

Secondary Antibodies (Target; Clone; Brand; Fluorochorme and corresponding catalog number)
goat anti-rabbit IgG; polyconal; Invitrogen; AF488 Cat#A11008
goat anti-rabbit IgG; polyconal; Invitrogen; AF594 Cat#A11072
goat anti-mouse IgG1; polyconal; Invitrogen; AF594; Cat#A21125

Western Blot
Primary Antibodies (Target; Clone; Brand; Fluorochorme and corresponding catalog number):
rabbit anti-human PPAR-γ; C26H12; Cell Signaling; unconjugated Cat#2435
rabbit anti-human pS6; D57.2.2E; Cell Signaling; unconjugated Cat#4858S
rabbit anti-human S6; 5G10; Cell Signaling; unconjugated Cat#2217S
mouse anti-human MCT1; H-1; Santa Cruz; unconjugated Cat#sc-365501
rabbit anti-human Histone H2B; polyclonal; Sigma-Aldrich; unconjugated Cat#SAB4502231

mouse anti-human F-Actin; ACTN05; Invitrogen; unconjugated Cat#MA5-11869
rabbit anti-human pAMPK; 40H9; Cell Signaling; unconjugated Cat#2535S

Secondary Antibodies (Target; Clone; Brand; Fluorochorme and corresponding catalog number):
goat anti-mouse IgG, polyclonal; Thermo Fisher; HRP Cat#G21040
goat anti-rabbit IgG; polyclonal; Fisher scientific; HRP Cat#31462

Validation

All antibodies were validated by the supplier (BioLegend, Cell Signaling, Novacastra, Dako, Santa Cruz; Invitrogen, Sigma-Aldrich, Thermo Fisher, Fisher scientific) and were checked in our lab by comparing to the manufacturer's or in-house results, using isotope controls.

Statement from BioLegend: Specificity testing of 1-3 target cell types with either single- or multi-color analysis (including positive and negative cell types). Once specificity is confirmed, each new lot must perform with similar intensity to the in-date reference lot. Brightness (MFI) is evaluated from both positive and negative populations. Each lot product is validated by QC testing with a series of titration dilutions.

Statment from Cell Signaling:
Our flow-validated products undergo rigorous testing in biologically relevant models, ensuring specificity and an optimal signal-to-noise ratio (S/N) for both conjugated and unconjugated antibodies. Cross-platform validation further confirms antibody specificity. In addition, all antibodies have been tested for optimal dilution, specificity, stability and lot-to-lot reproducibility to ensure they work the first time, every time.Our thoroughly validated antibodies for flow cytometry make it possible to examine complex intracellular signaling cascades in cell lines, dissociated tissues, aspirates, or hematology specimens, and trust the accuracy of your results.
Validation Includes:Use of positive and negative cell lines; Comparison of signal to isotype control to estimate nonspecific binding of primary antibodies; Treatment with pathway-specific inhibitors/activators; Treatment with blocking peptides, siRNA, and/or expression vectors; Phosphatase treatment to confirm phospho-specificity; Extensive quality control testing to guarantee stability over time and to eliminate lot-to-lot variability; Optimization of protocols and determination of optimal dilutions; Validation across multiple applications to confirm antibody specificity; The performance of our antibodies is routinely validated across multiple platforms.

Statement from Sigma-Aldrich:
It is common for commercial antibody developers to assess the on-target binding of an antibody during post immunization screening solely by Western blot (WB) or immunohistochemistry (IHC). This screen ensures that the antibody in development is, at this initial stage, recognizing the expected target and performing similarly to previous lots, if applicable. However, this simple screening is not adequate to assure application suitability and true lot-to-lot consistency.
We therefore subsequently test in as many additional immunodetection applications as practical in samples chosen to be relevant to the intended use of the product. These include immunohistochemistry, immunocytochemistry (ICC), Western blot, ELISA, immunoprecipitation, and more. This in-depth application testing can help assess the antibody’s specificity for the target and provides contextually relevant validation in applications and samples most likely to be used by our customers. This application-specific data should be reviewed by the researcher and appropriately assessed for the researchers intended use. Beyond review of the vendor generated application data, critical review of the epitope, species reactivity, clonality, appropriate host species, and development of appropriate controls are critical responsibilities of the researcher in the selection and use of antibody product.

Statement from ThermoFisher:
Thermo Fisher Scientific is committed to antibody performance and specificity testing. To support this commitment, each Invitrogen antibody that is indicated for western blotting applications has been tested using a protocol similar to that provided on our website. These tests help confirm antibody performance and help ensure superior results when used in experiments.

Statement from Novocastra:
Each antibody in the Novocastra HD range has been independently evaluated by an external QA scheme, in comparison with two market leading vendors. Each antibody within the HD portfolio has been optimized to deliver high quality staining, for results you can trust.

Flow Cytometry

Plots

Confirm that:

- [x] The axis labels state the marker and fluorochrome used (e.g. CD4-FITC).
- [x] The axis scales are clearly visible. Include numbers along axes only for bottom left plot of group (a 'group' is an analysis of identical markers).
- [x] All plots are contour plots with outliers or pseudocolor plots.
- [x] A numerical value for number of cells or percentage (with statistics) is provided.

Methodology

Sample preparation

Peripheral Blood Mononuclear Cells (PBMCs) were isolated according to the Standard Operating Procedure (SOP): PBMC Isolation using SepMate™.
CD4+ T cells were isolated from PBMCs using the EasySep positive selection kit (Stemcell Technologies) as per the manufacturer’s instructions. Positively selected CD4+ T cells were stained for the subsequent sorting of the TH cell subset.

Memory TH cell subsets were sorted with a purity of > 90% according to the expression of chemokine receptors from CD45RA-CD25-CD8-CD3+ cells: TH1 (CXCR3+CCR8-CCR6-CCR4-), TH2 (CXCR3-CCR8-CCR6-CCR4+), TH9 (CXCR3-CCR8+CCR6-CCR4+), and TH17 (CXCR3-CCR8-CCR6+CCR4+). Individual memory TH cells were directly sorted from CD4+ T cells into 96-well plates according to the expression of chemokine receptors. Individual cells were grown by periodic activation with phytohemagglutinin (1 ug/ml; Sigma-Aldrich), and irradiated allogeneic feeder cells (5×104 per well) in a culture medium. Half of the nutrient medium for T cell culture was replaced with a fresh medium every second day, starting from day 2 after reactivation. TH cell clones were analyzed in the resting state (≥ 14 days after the last expansion) or at different time points after polyclonal activation.
Human naïve T cells were isolated from PBMCs using the EasySep™ Human naïve CD4+ T Cell Isolation Kit (Stemcell Technologies) as per the manufacturer's instructions.
Naïve T cells were stimulated with aCD3/CD2/CD28 beads (T cell/bead = 2:1, Miltenyi) and primed into effector CD4+ T cell subsets with no addition of cytokines for TH0 cells, IL-12 (5 ng/ml) (BioLegend) for TH1 cells, IL-4 (50 ng/ml) (BioLegend) for TH2 cells, IL-4 (50 ng/ml) and TGF-b (5 ng/ml) (R&D Systems) for TH9 cells, and TGF-b (5 ng/ml) for iTREG. From cell culture initiation to analysis, the culture medium was supplemented with the indicated cytokines every other day.
Lesional and non-lesional skin biopsies of positive patch test reactions to different allergens were cultured in culture medium and respective treatments were added to the culture medium.
To analyze surface expression of proteins, T cells were washed twice with cell wash and stained at room temperature for 15 minutes with the respective markers. After staining, cells were washed twice with cell wash before acquisition.
To analyze cytokine production and S6 phosphorylation, T cells were polyclonally activated using ImmunoCult Human CD3/CD2/CD28 T Cell Activator (1:100) (Stemcell Technologies). Before activation and at different time points thereafter, T cells were additionally stimulated with PMA (50 ng/ml) (Sigma-Aldrich), ionomycin (1 uM) (Sigma-Aldrich), and brefeldin A (10 ug/ml) (Sigma-Aldrich) for 4 h. After viability and surface staining, the cells were fixed and permeabilized using Cytofix/Cytoperm kit (BD Biosciences) as per the manufacturer's instructions. Fluorescence-labeled antibodies were used to detect intracellular proteins, as well as phosphorylation.

Instrument

Flow cytometry data were collected on CytoFLEX (Beckman Coulter)

Software

CytExpert software version 2.4 (Beckman Coulter)

Cell population abundance

Cell population abundance varies between different donors. At least 5000 live T cell were collected by Flow cytometry for analysis. Memory TH cell subsets were sorted with a purity of > 90%.

Gating strategy

1. FACS sorting strategy of CD4+ T cell subsets isolated from human PBMCs: CD4+ T cells were stained for the subsequent sorting of TH cell subset. Memory TH cell subsets were sorted with a purity of > 90% according to the expression of chemokine receptors from CD45RA-CD25-CD8-CD3+ cells: TH1 (CXCR3+CCR8-CCR6-CCR4-), TH2 (CXCR3-CCR8-CCR6-CCR4+), TH9 (CXCR3-CCR8+CCR6-CCR4+), and TH17 (CXCR3-CCR8-CCR6+CCR4+).
2. Gating strategy for in vitro-primed TH cell subsets or FACS sorted in vivo-primed TH cells: Cells were gated based on FSC height and FSC area for singlets, either Zaq negative or SSC and FCS for live cells. Live cells were further analyzed for cytokine expression or phosphorylation of the target of interest.
3. Gating strategy for T-cells emigrating out of skin biopsies: Cells were gated based on FSC height and FSC area for singlets, SSC and FCS for lymphocytes, Zaq negative for live cells and further gated for CD3+ and CD4+ or CD8+ cells. CD4+ cells were analyzed for cytokine expression or phosphorylation of the target of interest.

☒ Tick this box to confirm that a figure exemplifying the gating strategy is provided in the Supplementary Information.
